# Trehalose Biosynthesis Gene *otsA* Protects against Stress in the Initial Infection Stage of *Burkholderia*-Bean Bug Symbiosis

**DOI:** 10.1128/spectrum.03510-22

**Published:** 2023-03-28

**Authors:** Junbeom Lee, Bohyun Jeong, Ha Ram Bae, Ho Am Jang, Jiyeun Kate Kim

**Affiliations:** a Metabolomics Research Center for Functional Materials, Kyungsung University, Busan, South Korea; b Department of Microbiology, Kosin University College of Medicine, Busan, South Korea; c Department of Biology, College of Natural Sciences, Soonchunhyang University, Asan, South Korea; University of Camerino

**Keywords:** *Burkholderia*, *Riptortus*, osmotic pressure, *otsA*, stress resistance, symbiosis, trehalose

## Abstract

Trehalose, a nonreducing disaccharide, functions as a stress protectant in many organisms, including bacteria. In symbioses involving bacteria, the bacteria have to overcome various stressors to associate with their hosts; thus, trehalose biosynthesis may be important for symbiotic bacteria. Here, we investigated the role of trehalose biosynthesis in the *Burkholderia*-bean bug symbiosis. Expression levels of two trehalose biosynthesis genes, *otsA* and *treS*, were elevated in symbiotic *Burkholderia insecticola* cells, and hence mutant Δ*otsA* and Δ*treS* strains were generated to examine the functions of these genes in symbiosis. An *in vivo* competition assay with the wild-type strain revealed that fewer Δ*otsA* cells, but not Δ*treS* cells, colonized the host symbiotic organ, the M4 midgut, than wild-type cells. The Δ*otsA* strain was susceptible to osmotic pressure generated by high salt or high sucrose concentrations, suggesting that the reduced symbiotic competitiveness of the Δ*otsA* strain was due to the loss of stress resistance. We further demonstrated that fewer Δ*otsA* cells infected the M4 midgut initially but that fifth-instar nymphs exhibited similar symbiont population size as the wild-type strain. Together, these results demonstrated that the stress resistance role of *otsA* is important for *B. insecticola* to overcome the stresses it encounters during passage through the midgut regions to M4 in the initial infection stage but plays no role in resistance to stresses inside the M4 midgut in the persistent stage.

**IMPORTANCE** Symbiotic bacteria have to overcome stressful conditions present in association with the host. In the *Burkholderia*-bean bug symbiosis, we speculated that a stress-resistant function of *Burkholderia* is important and that trehalose, known as a stress protectant, plays a role in the symbiotic association. Using *otsA*, the trehalose biosynthesis gene, and a mutant strain, we demonstrated that *otsA* confers *Burkholderia* with competitiveness when establishing a symbiotic association with bean bugs, especially playing a role in initial infection stage. *In vitro* assays revealed that *otsA* provides the resistance against osmotic stresses. Hemipteran insects, including bean bugs, feed on plant phloem sap, which may lead to high osmotic pressures in the midguts of hemipterans. Our results indicated that the stress-resistant role of *otsA* is important for *Burkholderia* to overcome the osmotic stresses present during the passage through midgut regions to reach the symbiotic organ.

## INTRODUCTION

Trehalose, α-d-glucopyranosyl-1,1-α-d-glucopyranoside, is a nonreducing disaccharide made of two glucose units linked by an α,α-1,1-glycosidic linkage (1). It is widespread in the biological world, serving multiple functions in a variety of organisms. In fungi, trehalose is involved in providing energy, protecting from stresses, and regulating metabolism, and threhalose-6-phosphate is a major signaling molecule (2). Trehalose acts as a stress protectant in primitive plants and a potential signal metabolite in higher plants (3). In insects, trehalose is present in hemolymph as a major blood sugar and is used as an instant source of energy during energy-requiring activities such as flight (4). In bacteria, there is less evidence that trehalose and trehalose-6-phosphate are involved in regulating metabolism or signaling. However, trehalose can serve as a carbon source and a stress protectant (1). Furthermore, in some bacterial species such as mycobacteria, it forms glycolipids, which are important components of the cell wall (5).

The role of trehalose as a stress protectant has been studied in various bacterial species. An early study showed that trehalose protected *Streptomyces* spores against heat and desiccation (6). In vegetative cells, trehalose is involved in resistance to desiccation, heat stress, and cold stress (7–11). In addition, its resistance role against osmotic stress has been studied in many bacteria (7, 9, 12–15). Stressors can induce denaturation of bacterial proteins and negatively affect the physiology of bacteria. Trehalose is known to prevent denaturation of proteins. The protective function of trehalose is due to its physical and chemical properties, which provide stability to cellular molecules (16).

When bacteria associate with a host to establish symbiosis, they are exposed to various stresses, such as osmotic pressure, hypoxia, nutrient depletion, and oxidative stress. Therefore, the ability to overcome these stressful conditions is important for establishment of a symbiotic relationship. In the *Bradyrhizobium*-soybean symbiosis, a high level of trehalose is crucial for Bradyrhizobium diazoefficiens to overcome hostile conditions, such as nutrient depletion and osmotic stress, during the early stages of the symbiotic association for root nodule development (13). Similarly, trehalose accumulation was observed in Bradyrhizobium japonicum and shown to support the development of root nodules on soybean plants (14). In the *Sinorhizobium*-alfalfa symbiosis, functional trehalose biosynthesis ensured that Sinorhizobium meliloti was symbiotically competitive (15). While the roles of trehalose in bacterium-plant symbiotic relationships have been reported, there are few studies of the roles of trehalose in bacterium-animal symbiotic relationships.

The *Burkholderia*-bean bug symbiosis is a well-established model of symbiosis for studying the molecular mechanisms underlying bacterium-insect symbiotic associations (17). The bean bug, *Riptortus pedestris* (*Hemiptera*: *Alydidae*), is a notorious pest in Southeast Asia. This insect has a specific symbiont, *Burkholderia insecticola*, that it harbors in its posterior midgut region, called the M4 (18). *Burkholderia* symbionts positively affect the biology of the host bean bug, conferring colonized hosts with larger body size, more egg production, and a stronger immune response to pathogens in comparison to aposymbiotic insects (17, 19, 20). Interestingly, this beneficial symbiont is not vertically transmitted but acquired from the environment every generation (21). In the laboratory, second-instar bean bugs have been inoculated with *B. insecticola* cells via drinking water to establish symbiosis. *B. insecticola* can be genetically manipulated and used to establish symbiosis with the host to investigate the molecular mechanisms underlying symbiotic associations (22). Several characteristics of *B. insecticola* have been identified as important for symbiosis, including flagellar motility, which enables *B. insecticola* to reach the M4, polyhydoxyalkanoate (PHA) biosynthesis, and biofilm formation, where both PHA and the biofilm support the persistence of symbionts in the M4 (23–27). Evaluation of the lipopolysaccharide (LPS) composition of *B. insecticola* has revealed that the structure of LPS changes upon symbiosis with bean bugs, that the LPS O-antigen enhances initial colonization by *B. insecticola* of the M4, and that the LPS core oligosaccharides are crucial for maintaining a symbiont population in the M4 (28–30). Because some of these bacterial symbiotic factors have a survival function under stressful conditions, we speculated that *B. insecticola* faces stressful conditions during the course of infection from the insect’s mouth (stylet) to midgut regions (M1, M2, and M3), followed by the symbiotic organ M4 midgut.

In this study, we hypothesized that trehalose biosynthesis might function to allow *B. insecticola* to overcome various stresses in the *Burkholderia*-bean bug symbiosis. First, we examined the expression of trehalose biosynthesis genes in *B. insecticola* symbionts and observed that two genes were expressed at higher levels in symbiotic bacteria than in cultured bacteria. Using mutant strains with defects in trehalose biosynthesis, we demonstrated that the *otsA* gene of *B. insecticola* is an important symbiotic factor for the initial colonization of the M4 midgut.

## RESULTS

### Symbiotic *Burkholderia* expresses higher levels of trehalose biosynthesis genes *otsA* and *treS* than cultured cells.

**FIG 1:**
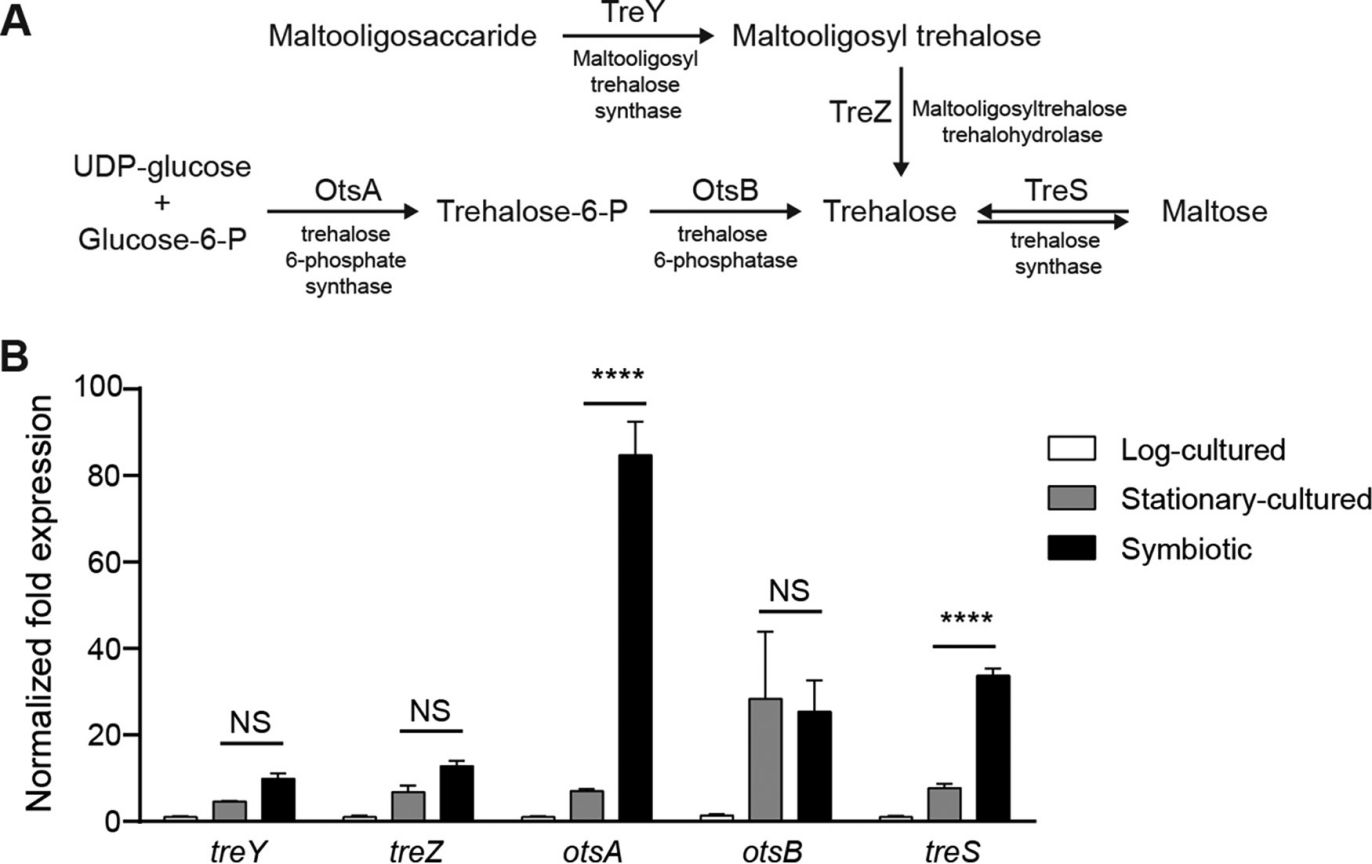
Trehalose biosynthesis genes and their expression in cultured and symbiotic *Burkholderia* cells. (A) Three trehalose biosynthetic pathways, the OtsA-OtsB, TreY-TreZ, and TreS pathways, are present in *B. insecticola*. (B) Expression of biosynthesis genes was compared among cultured cells in log and stationary phases and symbiotic cells. Means and SDs (*n* = 3) are shown as columns and error bars, respectively. Asterisks indicate statistically significant differences (two-way ANOVA with Dunnett’s *post hoc* test): NS, not significant; ****, *P* < 0.0001.

In *B. insecticola*, there are three trehalose biosynthetic pathways, involving OtsA and OtsB, TreY and TreZ, or TreS (Fig. 1A). OtsA with OtsB is the major pathway and consists of trehalose-6-phosphate synthase (OtsA), which catalyzes the reaction between glucose-6-phosphate and UDP-glucose to generate trehalose-6-phosphate, and trehalose-6-phosphate phosphatase (OtsB), which converts trehalose-6-phosphate to trehalose and inorganic phosphate. An alternative trehalose biosynthetic pathway is the TreY-TreZ pathway, which produces trehalose from maltooligosaccharides with the aid of maltooligosyltrehalose synthase (TreY) and maltooligosyltrehalose trehalohydrolase (TreZ). The third pathway involves TreS, trehalose synthase, which reversibly convert maltose to trehalose. *B. insecticola* possesses single copies of *otsB* (Locus tag, BRPE64_ACDS19500), *treY* (BRPE64_BCDS12550), *treZ* (BRPE64_BCDS12530), and *treS* (BRPE64_BCDS12500), but three paralogs of *otsA* (BRPE64_ACDS08420, BRPE64_ACDS13990, and BRPE64_ACDS19490).

To examine changes in the expression of trehalose biosynthesis genes in *B. insecticola* upon symbiotic association with the host insect, expression levels of *otsA*, *otsB*, *treY*, *treZ*, and *treS* genes were compared between symbiotic cells isolated from the M4 and cultured cells in the exponential and stationary phases (Fig. 1B). Because trehalose biosynthesis genes are expressed at higher levels during the stationary phase than the exponential phase in cultured cells, the expression of stationary-phase cells was set as the control for the multiple comparisons (two-way analysis of variance [ANOVA] with Dunnett’s *post hoc* test). The *otsB* expression of symbiotic cells was similar to that of stationary-phase cultured cells. Expression levels of *treY* and *treZ* in symbiotic cells were about 2-fold higher than those in stationary cultured cells. Levels of *otsA* (BRPE64_ACDS08420) were 12-fold higher in symbiotic cells than cultured cells. The other two *otsA* paralogs (BRPE64_ACDS13990 and BRPE64_ACDS19490) were similarly expressed in symbiotic and cultured cells (data not shown). Expression of *treS* was 4-fold higher levels in symbiotic cells than cultured cells. Based on these results, we focused on *otsA* and *treS* as symbiotic factor candidates to determine the role of trehalose biosynthesis in the *Burkholderia*-bean bug symbiosis.

### The *otsA* deletion mutant strain failed to have symbiotic competitiveness against the wild-type strain.

**FIG 2:**
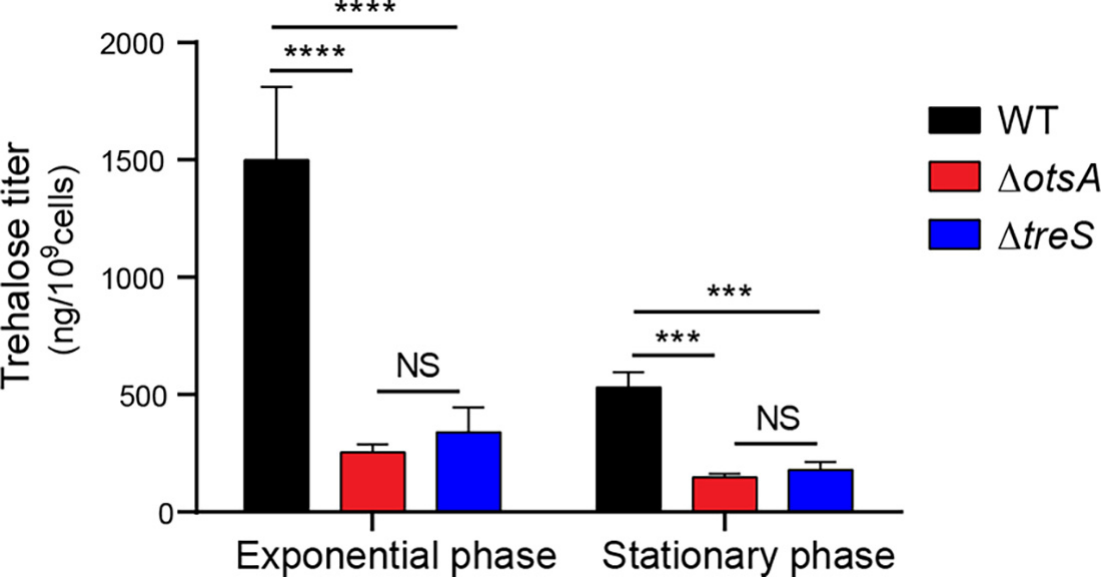
Trehalose titers of wild-type, Δ*otsA*, and Δ*treS* strains. Deletion mutant strains of the trehalose biosynthesis genes, *otsA* and *treS*, exhibited significantly lower trehalose titers (in nanagrams per 10^9^ cells) than the wild-type (WT) strain. Means and SD (*n* = 6) are shown as columns and error bars, respectively. Asterisks indicate statistically significant differences (two-way ANOVA with Tukey’s *post hoc* test): NS, not significant; ***, *P* < 0.001; ****, *P* < 0.0001.

To examine the roles of the trehalose biosynthesis genes *otsA* and *treS* in the *Burkholderia*-bean bug symbiosis, in-frame deletion mutant strains were generated. As shown in Fig. 2, trehalose levels in Δ*otsA* and Δ*treS* strains were significantly lower than those of the wild-type strain in both exponential and stationary phases. In particular, trehalose levels of Δ*otsA* and Δ*treS* in the exponential phase were about 6-fold and 4-fold lower than levels in the wild-type strain, respectively.

**FIG 3:**
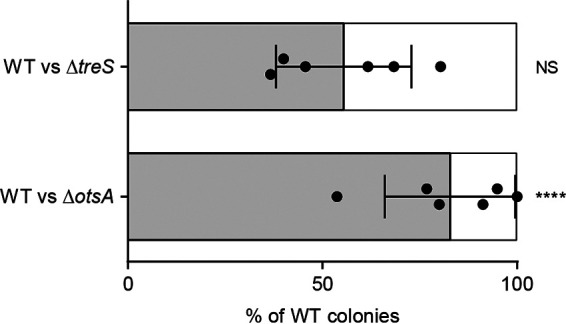
*In vivo* competition assays between wild-type and trehalose biosynthesis mutant strains. Symbiotic competitiveness of mutant strains was compared with that of the wild-type strain by inoculating bean bugs with a 1:1 bacterial solution (wild-type:mutant) followed by determination of the proportions of the strains in the fifth-instar M4. Upper bar graph indicates competition between the wild-type and Δ*treS* strains; lower bar graph indicates competition between the wild-type and Δ*otsA* strains. Each data point represents the percentage of wild-type colonies from an infected insect. Means and SD are shown as gray columns and error bars, respectively. An unpaired *t* test was used to evaluate the statistical significance of differences among groups: NS, not significant; ****, *P* < 0.0001.

To investigate the effect of deletion of trehalose biosynthesis genes on the *Burkholderia*-bean bug symbiosis, the competitiveness of Δ*otsA* and Δ*treS* strains was investigated by *in vivo* competition assay, where the mutant strain competed with the wild-type strain to associate with the insect host. Bean bugs were inoculated with a 1:1 bacterial solution of wild-type:mutant strains during the second-instar stage. At the fifth-instar stage, we measured the percentage of wild-type cells and mutant cells present in the M4 midgut. Both the wild-type and Δ*treS* strains had a similar colonization rate, indicating that Δ*treS* was not competitively inferior to the wild-type strain. However, the Δ*otsA* strain exhibited significant impairment of symbiotic competitiveness, with 8-fold less (11.7 ± 4.6%; mean ± standard deviation [SD]) colonization of the M4 midgut than the wild-type strain (88.3 ± 4.6%) (Fig. 3). These results indicated that *otsA*, but not *treS*, confers *B. insecticola* with competitiveness when establishing a symbiotic association with bean bugs.

### The *otsA* deletion mutant strain was susceptible to osmotic stresses.

**FIG 4:**
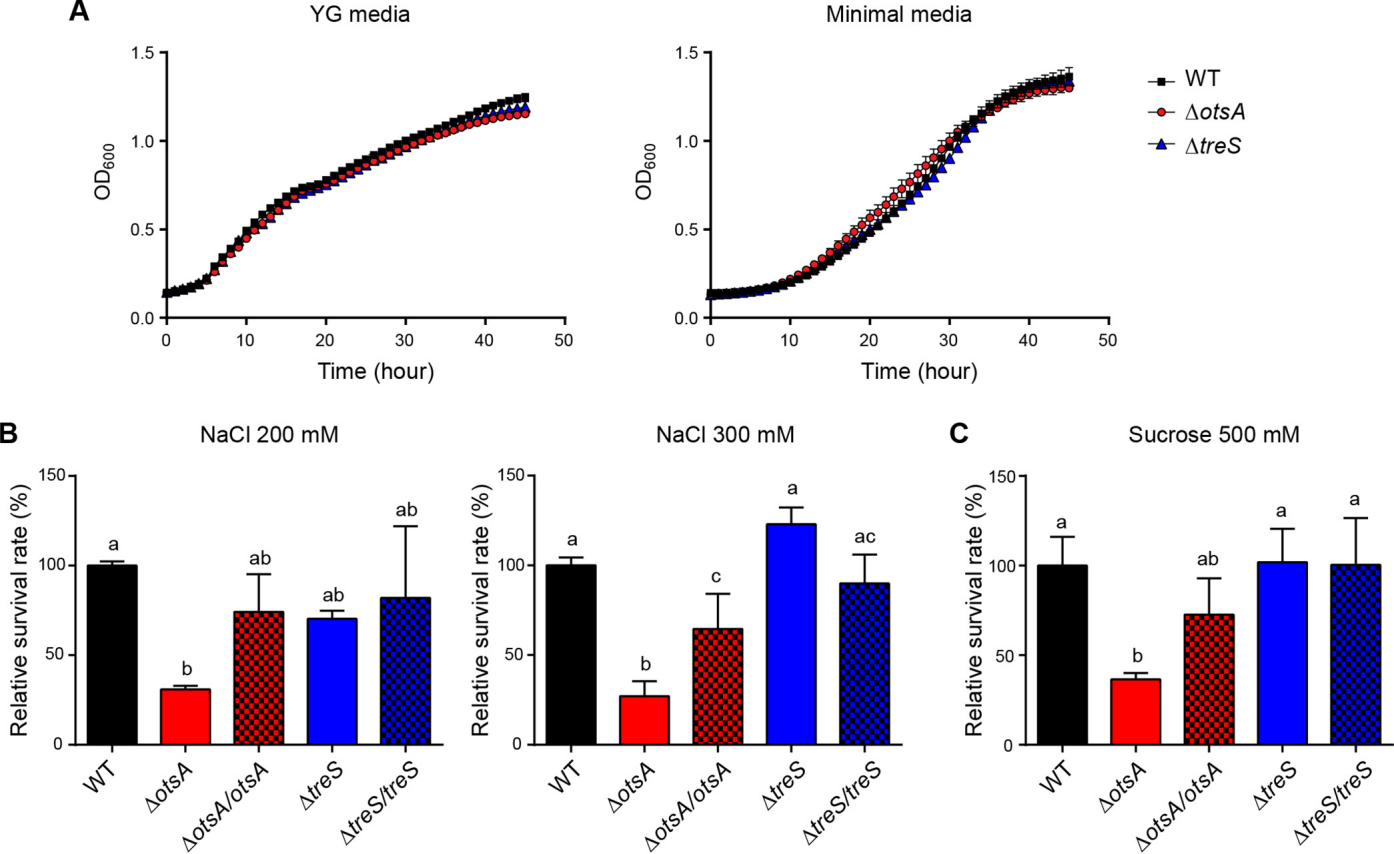
Growth and survival of wild-type, Δ*otsA*, and Δ*treS* strains under stressful conditions. (A) Growth curves of wild-type, Δ*otsA*, and Δ*treS* strains did not differ in nutrition-rich YG medium or nutrient-minimal M9 medium. (B and C) Survival rates of mutant strains (Δ*otsA* and Δ*treS*) and complemented strains (Δ*otsA/otsA* and Δ*treS/treS*) were compared with those of the wild-type strain under high-salt conditions (200 mM and 300 mM NaCl) (B) and a high-sucrose condition (500 mM sucrose) (C). Means and SD (*n* = 3) are shown as columns and error bars. Different letters (a, b, c) on the top of the columns indicate statistically significant differences (*P* < 0.05; one-way ANOVA with Tukey’s *post hoc* test). The data shown here are representative of three independent experiments.

To understand the symbiotic functions of *otsA*, the *in vitro* characteristics of the *B. insecticola* Δ*otsA* strain were examined using various stress assays. We selected physiologically relevant stresses that *B. insecticola* might encounter in the process of symbiotic association with host bean bugs. These included growth under minimal nutrient conditions, osmotic pressure due to high salt and/or high sucrose concentrations, presence of surfactant, and oxidative stresses. We also evaluated motility, which is critical for reaching the M4, as well as biofilm formation, which is a persistence factor for M4 colonization. Growth curves of Δ*otsA* and Δ*treS* strains were similar to those of the wild-type strain in both nutrient-rich yeast extract-glucose (YG) medium and nutrient-minimal M9 medium, indicating that mutation of these genes did not affect bacterial growth in these media (Fig. 4A). Δ*otsA* and Δ*treS* strains did not differ from the wild-type strain in terms of resistance against surfactant and oxidative stresses as assessed by sodium dodecyl sulfate and hydrogen peroxide treatment, respectively (see Fig. S1 in the supplemental material). Furthermore, motility and biofilm formation were similar among wild-type, Δ*otsA*, and Δ*treS* strains (Fig. S2). However, the Δ*otsA* strain showed a dramatic increase in susceptibility to the osmotic stresses generated by high salt and sucrose concentrations in comparison to the wild-type and Δ*treS* strains. Survival rates of the Δ*otsA* strain were 30.9 ± 2.0% and 27.1 ± 8.3% of those of the wild-type strain when treated with 200 mM and 300 mM NaCl, respectively (Fig. 4B). Treatment with 500 mM sucrose resulted in decreased survival of the Δ*otsA* strain relative to the wild-type strain (36.5 ± 3.5% of that of wild-type strain) (Fig. 4C). The complemented Δ*otsA/otsA* strain showed some restoration of the survival rates under these stress conditions. These findings suggested that *otsA* is a symbiotic factor that provides the resistance against osmotic stresses. We verified the expression levels of trehalose biosynthesis genes under stressful conditions. These genes exhibited higher levels of expression under osmotic stresses than in the absence of stresses (Fig. S3).

### *otsA* is required for *Burkholderia* cells to migrate to the M4 midgut during the initial infection stage.

Next, we aimed to determine in which stage of the symbiotic association the stress resistance function of *otsA* plays a role. As shown in Fig. 3, a low number of Δ*otsA* cells were found in M4 when in the competition with the wild-type strain. This could have been due to decreased migration to the M4 and/or lower survival in the M4 of Δ*otsA* cells compared to wild-type cells. Therefore, we divided the symbiotic association into two stages: an initial infection stage, where bacteria migrate from the bean bug’s mouth to the M4, and a persistent stage, where bacteria colonize and persist in the M4 midgut.

**FIG 5:**
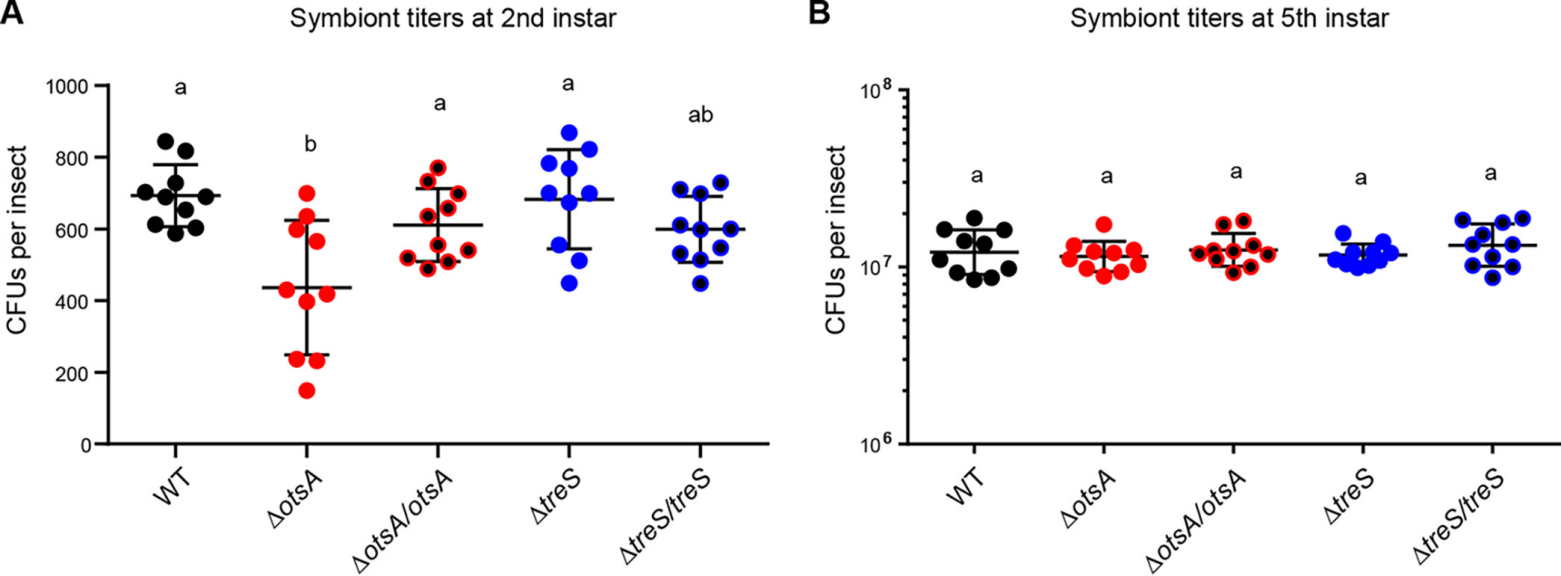
Symbiont titers of wild-type, Δ*otsA*, Δ*treS*, and complemented strains in the M4 symbiotic midgut. (A) Initial infection efficiency of the strains was analyzed by measuring symbiont titers of second-instar nymphs at 6 h postinoculation. (B) Bacterial persistence was analyzed by evaluating symbiont titers of fifth-instar nymphs. Symbiont titers were measured by culturing M4 homogenates and counting CFU of *B. insecticola*. Each data point represents CFU per insect. Means and SD are shown as horizontal lines and error bars, respectively. Different letters (a, b) indicate statistically significant differences (*P* < 0.05; one-way ANOVA with Tukey’s *post hoc* test).

To evaluate the role of *otsA* in the initial infection stage, CFU of *B. insecticola* cells arriving at the M4 were counted. We chose the 6-h postinoculation time point to collect the second-instar M4 midgut, as a previous study had reported that *B. insecticola* started to colonize the M4 around 6 h postinoculation (18). CFU counts of the Δ*otsA* strain present in the M4 were significantly lower than those of the wild-type strain at 6 h postinoculation, while CFU counts of the Δ*treS* strain were not significantly different from those of the wild-type strain or the complemented strains (Fig. 5A). Regardless of the number of bacteria arriving at the M4 midgut, the symbiont population reaches a maximum during the third instar, unless there is a defect in symbiotic adaptation in *B. insecticola* (21, 23). Therefore, the role of *otsA* in the persistent stage was examined by analyzing the Δ*otsA* symbiont population in fifth-instar M4 midguts. As shown in Fig. 5B, CFU counts of Δ*otsA* in the fifth-instar M4 were similar to those of the wild-type and Δ*treS* strains, indicating no defect in symbiotic adaptation to the M4 midgut. Consequently, there were no differences in growth rate or body weight between wild-type symbiotic insects and mutant (Δ*otsA* or Δ*treS*) symbiotic insects (Fig. S4). In summary, the Δ*otsA* strain exhibited reduced infectivity in the initial infection stage but had a normal symbiont population in the persistent stage. These results indicated that *otsA* plays a role in protecting *Burkholderia* cells against the stressful conditions they encounter during passage into the M4 midgut.

## DISCUSSIONS

One of a the key genes in the trehalose biosynthetic pathway is the *otsA* gene. Given that trehalose plays a role in stress resistance in many organisms, we investigated its role in the *Burkholderia*-bean bug symbiotic association. When we infected bean bugs with wild-type and Δ*otsA* strains simultaneously, the *otsA*-deficient strain failed to colonize the symbiotic M4 midgut compared to the wild-type strain, clearly indicating that *otsA* plays a role in the *Burkholderia*-bean bug symbiosis. To determine the symbiotic function of *otsA*, we performed *in vitro* assays using an Δ*otsA* strain and found that *otsA* is important for resistance to osmotic pressure. By *in vivo* monoinfection of bean bugs with the Δ*otsA* strain, we further identified that *otsA* plays an important role in the initial infection stage of symbiosis. At 6 h postinoculation, there were fewer Δ*otsA* cells in the M4 midgut than wild-type cells. However, the symbiont densities of these two strains in fifth-instar nymphs were similar, showing that *otsA* is not essential for the persistent stage in the M4 midgut. Our results indicated that *otsA* protects *B. insecticola* cells from stressors that the bacteria encounter during passage from the insect mouth to the M4, rather than stressors encountered inside the M4 midgut.

The trehalose synthase gene *treS* was expressed at 4-fold-higher levels in symbiotic *B. insecticola* cells than cultured cells (Fig. 1B). However, the Δ*treS* mutant strain showed neither increased susceptibility to *in vitro* stress assays nor impairment of *in vivo* symbiotic association. The lack of observation of a mutant phenotype may have been due to the reversible function of TreS, which converts maltose to trehalose and vice versa. In contrast, OtsA catalyzes the unidirectional reaction utilizing UDP-glucose and glucose-6-phosphate to synthesize trehalose-6-phosphate, which is subsequently converted into trehalose by OtsB (31). In many organisms, including bacteria, fungi, insects, and plants, the OtsA-OtsB pathway is the principal trehalose biosynthesis pathway (32–35). In this study, the *otsA* mutant strain clearly showed defects in stress resistance and symbiotic association, supporting that the OtsA-OtsB pathway is the major trehalose biosynthetic pathway in *B. insecticola*.

While the role of trehalose biosynthesis in bacterium-animal symbioses has not been studied, the role of trehalose has been investigated in several pathogens. In Escherichia coli, the OtsA-OtsB pathway is regulated by the stress response regulator RpoS and provides resistance to heat and cold shock (10, 36). In Salmonella enterica, an *otsA* mutant strain was more susceptible to heat and osmotic stresses but showed no defects in bacterial colonization in mouse spleen tissues after oral infection compared to the wild-type strain (7). Expression of the *otsBA* operon was induced by high osmolarity and temperature in Acinetobacter baumannii. Trehalose biosynthesis was abolished by deletion of the *otsB* gene, and A. baumannii with the *otsB* deletion was susceptible to heat and lost its infectivity toward Galleria mellonella larvae (9, 37). In the plant pathogen Ralstonia solanacearum, Δ*otsA* and Δ*treY* Δ*treS* Δ*otsA* mutant strains were susceptible to osmotic stress and were significantly less virulent than the wild-type strain in tomato infection (12). A study of Xanthomonas citri, which causes citrus canker, demonstrated by *otsA* deletion that *otsA* played a role in resistance to salt and oxidative stresses and colonization of plant tissues (38). In addition to its role in stress resistance, trehalose is a component of cell walls of Mycobacterium, *Nocardia*, and *Corynebacterium* species, where it couples with lipids. The best known example of trehalose lipids is trehalose-6,6-dimycolate (TDM, or cord factor) in Mycobacterium tuberculosis (39). The OtsA-OtsB pathway is required for M. tuberculosis growth and for its virulence in a mouse model (40).

In this study, we elucidated the role of *otsA* in a bacterium-insect symbiosis for the first time. Expression of *otsA* contributed to symbiotic association with the host bean bug. In particular, *otsA* played a role in the initial infection stage during which bacteria taken up orally migrated through the midgut regions (M1, M2, and M3) to colonize the symbiotic organ M4. Previous studies have suggested that the midgut of insects is a harsh environment for bacteria due to anoxic and slightly acidic conditions (41, 42). Hemipteran insects, including bean bugs, pierce plants with their stylet to feed on plant phloem sap. Plant sap is sucrose-rich with sucrose concentrations ranging from 0.2 to 1.5 mol/liter, which is known to affect the osmotic pressure of hemolymph (43). It is likely that the high osmolality of phloem sap leads to high osmotic pressure in the midguts of hemipterans. Based on our results, we speculate that osmotic stresses in the midgut regions of M1, M2, and M3 of bean bugs may reduce the initial colonization by *otsA*-deficient *B. insecticola* of the M4 midgut.

Even though *otsA* did not appear to play an essential role in the persistent stage, it was still expressed at 10-fold-higher levels in symbiotic cells than in cultured cells. It would be worthwhile to address trehalose degradative pathways in *B. insecticola* and their symbiotic roles in the *Burkholderia*-bean bug symbiosis in future studies.

## MATERIALS AND METHODS

### Bacteria, plasmids, and media.

Bacterial strains and plasmids used in this study are listed in Table S1. Escherichia coli strains were cultured in LB medium (1% [wt/vol] tryptone, 0.5% [wt/vol] yeast extract, and 0.5% [wt/vol] NaCl) at 37°C. *B. insecticola* strains were cultured in YG medium (0.5% [wt/vol] yeast extract, 0.4% [wt/vol] glucose, and 0.1% [wt/vol] NaCl) at 27°C. Antibiotic concentrations were as follows: 30 μg/mL for rifampin, 50 μg/mL for kanamycin, and 30 μg/mL for chloramphenicol.

### Measurement of expression of trehalose biosynthesis genes by quantitative PCR.

Symbiotic cells were isolated from the M4 midgut as described previously (44), and stationary-phase cultured cells and log-phase cultured cells were prepared to have 2 × 10^8^ cells per sample. Bacterial samples were treated with RNAprotect bacteria reagent (Qiagen Inc., Valencia, CA, USA) for 5 min at room temperature and centrifuged at 5,000 × *g* for 10 min to collect the bacterial pellet. Cell pellets were resuspended and incubated in 100 μL of nuclease-free water containing 100 μg/mL proteinase K and 400 μg/mL lysozyme for 10 min at room temperature. Then, preheated RiboEx (GeneAll Biotechnology, Seoul, South Korea) was added to these samples, and bacterial RNA was extracted according to the manufacturer’s instructions. RNase-free DNase I (Illumina, San Diego, CA, USA) was added to the extracted RNA. After DNase treatment for 15 min at 37°C, samples were repurified with RiboEx. Reverse transcription of the RNA samples was performed using TOPscript RT DryMIX containing random hexamer primers (Enzynomics, Daejeon, South Korea). The generated cDNA was subjected to quantitative PCR (qPCR) using TOPreal qPCR 2× PreMIX with SYBR green (Enzynomics). Primer sequences used in qPCR are listed in Table S2. The PCR temperature profile comprised 1 cycle of 95°C for 10 min followed by 40 cycles of 95°C for 10 s, 60°C for 15 s, and 72°C for 20 s, using a CFX96 real-time PCR system (Bio-Rad, Hercules, CA, USA). The comparative threshold cycle (ΔΔ*C_T_*) method was used to analyze relative gene expression levels, and the *recA* gene (BRPE64_ACDS04710) of *B. insecticola* was used as an endogenous control gene.

### Measurement of trehalose concentration by LC-MS/MS.

Stationary-phase cultured cells and log-phase cultured cells were prepared to have 1 × 10^9^ cells per sample, and bacterial samples were frozen in liquid nitrogen and stored in a −80°C freezer to quench metabolic activity. Metabolites were extracted from bacterial samples with 75% (wt/vol) acetonitrile solvent by sonication and heat treatment and filtered through a 0.22-μm polyvinylidene difluoride membrane to be used for liquid chromatography with tandem mass spectrometry (LC-MS/MS) analysis. LC-MS/MS was performed at the Metabolomics Research Center for Functional Materials (Kyungsung University, Busan, South Korea). Briefly, high-performance LC was performed using an Agilent 1260 system (Agilent Technologies Inc., Santa Clara, CA, USA) with a Phenomenex NH2 column (4.6 mm by 250 mm, 5 μm, 100 Å). Binary mobile phase for analytical separation consisted of 75% acetonitrile with 0.1% formic acid for 20 min. Samples (1 μL) were injected at a flow rate of 1 mL/min and a column temperature of 40°C. The detector used was an AB Sciex 4500 triple quadrupole mass spectrometer (AB Sciex, Foster City, CA, USA) operating in multiple reaction monitoring mode. Samples were ionized via electrospray ionization (ESI) in negative mode. The ESI source was operated with an ion spray voltage of −4,000 V and heater temperature of 400°C. Gas settings were as follows: curtain gas 20, collision gas 9, ion source gas 20. Unit mass resolution was used in Q1 and Q3 to select the following selected reaction monitoring transitions (with the collision energy [CE] given in parentheses): *m/z* 387.0 → 59.0 (CE, −32 eV) for trehalose. To determine the concentration of trehalose, pure trehalose was purchased from Sigma-Aldrich and solubilized in 1 mL of pure water. A standard calibration curve was generated using 2-fold serial dilutions of 250-ng/mL trehalose in acetonitrile.

### Generation of mutant strains.

Deletion of genes of interest was accomplished by homologous recombination followed by allelic exchange using the pK18mobsacB suicide vector containing the 5′ and 3′ regions of the gene of interest, as described previously (45). The 5′ and 3′ regions of *otsA* and *treS* were first amplified from *B. insecticola* RPE75 by PCR using the primers listed in Table S2. After PCR, fragments of the 5′ and 3′ regions of the gene were ligated to pK18mobsacB; this vector was transformed into E. coli DH5α cells. Then, triparental conjugation was performed to transfer the cloned pK18mobsacB vector to recipient RPE75 cells with conjugal help from E. coli HBL1 cells (Table S1). *B. insecticola* cells with the first crossover were selected on YG agar plates containing rifampin and kanamycin. Single-crossover cells were cultured in YG medium without kanamycin to allow the second crossover. Cells with a double crossover were selected on YG agar plates containing rifampin and sucrose (200 μg/mL). Deletion mutant strains obtained by double crossover were identified by PCR.

### Generation of complemented mutant strains.

To complement the *otsA* or *treS* deletion mutants, the *otsA* or *treS* genes were cloned using the broad-host-range vector pBBR122, as described previously (27). The blunt-end PCR inserts containing the *otsA* or *treS* gene were prepared using the primers *otsA*-com-P1 and P2 or *treS*-com-P1 and P2, respectively (Table S2). The amplified DNA fragments were cloned into the DraI site of pBBR122 and transformed to E. coli DH5α cells. Using triparental conjugation with E. coli HBL1, pBBR122 carrying the *otsA* or *treS* gene was transferred to the recipient *Burkholderia* Δ*otsA* or Δ*treS* mutant strain, respectively. The complemented clones were selected on YG agar plates containing rifampin and kanamycin.

### *In vivo* competition assay.

To test the competitiveness of the mutant strains in the symbiotic association, a mixed-inoculum solution (10^7^ cells/mL) of wild-type and mutant cells (1:1 ratio) was prepared and provided to second-instar nymphs. At the fifth-instar nymphal stage, symbiotic *B. insecticola* cells were isolated from the M4 midgut and spread on YG agar plates with rifampin. Sixty colonies were randomly selected from each insect and examined for *otsA* or *treS* deletion by PCR (Table S2).

### Measurement of bacterial growth in liquid media.

Growth patterns of *B. insecticola* strains in YG medium or in M9 minimal medium (1.3% Na_2_HPO_4_·2H_2_O, 0.3% KH_2_PO_4_, 0.1% NH_4_Cl, 0.1% NaCl, 100 mM CaCl_2_, 100 mM MgSO_4_, 0.4% glucose) were examined at 27°C for 45 h. Stationary-phase cells were prepared to have an optical density at a wavelength of 600 nm (OD_600_) of 0.02 as a starting solution. During cultivation, the OD_600_ of bacterial cell solutions was measured every hour using a Tecan Infinite M200 plate reader (Tecan Group Ltd., Männedorf, Switzerland).

### *In vitro* stress assays.

*B. insecticola* strains at exponential phase grown in YG medium were washed with 10 mM phosphate buffer (pH 7.0; PB) and adjusted to have 1 × 10^7^ cells/mL. For the salt stress assay, cells were incubated with 200 mM or 300 mM NaCl in PB for 18 h at room temperature before being spread on YG agar plates. For the osmotic stress assay, cells were incubated in 500 mM sucrose in PB for 18 h at room temperature. Cells incubated in PB for 18 h at room temperature were used as controls. After the incubation, cells were diluted and plated on YG agar plates, and the number of CFU was determined. The survival rate was calculated as follows: (CFU_stress_/CFU_control_) × 100%. The relative survival rates of mutant strains were further calculated by comparing them with the survival rate of the wild-type strain: (survival rate_mutant_/survival rate_WT_) × 100%.

### Insect rearing and symbiont inoculation.

Bean bugs were reared at 27°C under a long day cycle of 16 h light and 8 h dark in clear plastic cages supplied with soybean seeds and drinking water (distilled water containing 0.05% ascorbic acid; DWA) as described previously (32). When newborn nymphs molted into the second instar, DWA was removed from the cages for 6 h, and then inoculum solution (DWA containing 10^7^ cells *B. insecticola* strain/mL) was provided to the nymphs for 1 day to facilitate symbiotic association.

### CFU assay to estimate symbiont titer.

To investigate *B. insecticola* CFU in the initial infection stage, newly molted second-instar nymphs were infected with a symbiont inoculum solution consisting of log-phase cells suspended in DWA at a concentration of 10^8^ cells/mL. At the 6-h postinoculation time point, M4 midguts dissected from individual insects were separately collected in 100 μL of PB and homogenized by pipetting followed by spreading on YG agar plates with rifampin. After 2 days of incubation at 30°C, colonies on the plates were counted.

To investigate bacterial CFU in the persistent stage, second-instar nymphs were infected with 10^7^ cells/mL inoculum solution. At the fifth-instar nymphal stage, the M4 midguts were dissected and collected individually in 100 μL PB. Dissected M4 midgut samples were homogenized with a plastic pestle and serially diluted in PB. Diluted samples were spread on YG agar plates with rifampin. After 2 days of incubation at 30°C, CFU were counted. Symbiont titers per insect were calculated by multiplying the counted CFU by the dilution factor.

### Statistical analyses.

The statistical significance of differences among groups was determined by unpaired *t* tests and one-way or two-way ANOVA with multiple-comparison tests using GraphPad Prism version 6.07 (GraphPad Software, San Diego, CA, USA).

### Data availability.

The data sets generated and analyzed during the current study are available from the corresponding author on reasonable request.
