# Supplementary material for: Trehalose Biosynthesis Gene otsA Protects against Stress in the Initial Infection Stage of Burkholderia-Bean Bug Symbiosis
Source: Microbiol Spectr. 2023 Mar 28;11(2):e03510-22. doi: 10.1128/spectrum.03510-22 (PMC10100943; doi:10.1128/spectrum.03510-22)

Supplementary Information

Trehalose biosynthesis gene *otsA* protects against stress in the initial infection stage of the *Burkholderia*-bean bug symbiosis

Junbeom Lee, Bohyun Jeong, Ha Ram Bae, Ho Am Jang, Jiyeun Kate Kim

SUPPLEMENTARY METHODS

Disk diffusion assay. Susceptibility of *Burkholderia insecticola* to surfactant and oxidative stress was measured by disk diffusion assay. *B. insecticola* cells were cultured to the end of log phase (OD_{600}, 0.1). One milliliter of cultured cell solution was mixed with 50 ml of unsolidified YG-agar media to achieve a final OD_{600} of 0.02. Three milliliters of cell-YG-agar solution was poured into 35 mm dishes and allowed to solidify. For the surfactant assay, different concentrations of sodium dodecyl sulfate (SDS) were applied to disks placed in the middle of the dishes. For the oxidative stress assay, different concentrations of hydrogen peroxide (H_2O_2) were applied to the disks. After incubating dishes at 30°C for 24 h, the diameters of inhibition zones were measured.

Motility assay. Five microliters of mid-log *B. insecticola* cells (OD_{600}, 0.3) was injected into the middle of soft YG-agar plate prepared with 0.2% agar. Plates were cultured for 48 h, and the diameters of swimming zones were measured.

Microtiter plate biofilm assay. Mid-log phase *B. insecticola* strains were prepared by adjusting the OD_{600} to 0.8 in YG medium with appropriate antibiotics, and 150 μl of the cell solution was added to each well of a 96-well plate. The 96-well plate was incubated at 30°C for 48 h with shaking at 150 rpm. At the end of the incubation, OD_{600} values of each well were measured using a TECAN Infinite M200 plate reader. First, 96-well plates were washed three times with PB, and adherent biofilms were fixed with 99% methanol for 10 min. After removing the methanol and air-drying, 200 μl of a 0.1% crystal violet solution was added to each well. A blank control was prepared by adding 150 μl of a crystal violet solution to the wells. After incubating for 20 min, the crystal violet solution was removed, and the wells were washed thoroughly with tap water and air-dried. Biofilm-staining crystal violet was solubilized in 200 μl of 30% acetic acid, and the OD_{540} of each well solution was measured using a using a TECAN Infinite M200 plate reader.

Measurements of insect growth and fitness. Adult emergence rate was monitored by inspecting late fifth instar nymphs and counting the number of newly molted adult insects every day. On the second day after molting to an adult, early adult insects were anesthetized with CO_2, immersed in acetone for 5 min, and then completely dried by incubation in a 70°C oven, after which dry body weight was measured.

Quantitative PCR of trehalose biosynthesis genes under stress conditions. To measure the expression of trehalose biosynthesis genes in response to stress, stationary phase *B. insecticola* WT was cultured in YG medium supplemented with 300 mM NaCl and 500 mM sucrose until the exponential phase. Bacterial samples of 2 x 10^8 cells per sample were treated with RNAprotect bacteria reagent (Qiagen Inc., Valencia, CA, USA), and subsequent experiments followed the order specified in the Materials and Methods section of the main manuscript.

Table S1. Bacterial strains and plasmids used in this study.

Bacterial strains or plasmids	Characteristics	Reference[a]
Burkholderia insecticola		
RPE75	*B. insecticola* (RPE64); Rif^R	[1]
BKJ002	RPE75 *ΔotsA*; Rif^R	This study
BKJ003	RPE75 *ΔtreS*; Rif^R	This study
Escherichia coli		
DH5α	F–Φ80*lac*ZΔM15 Δ(*lac*ZYA-*arg*F) U169 *rec*A1 *end*A1 *hsd*R17 (rK–, mK+) *pho*A *sup*E44 λ– *thi*-1 *gyr*A96 *rel*A1	[2]
PIR1	F- *Δlac*169 *rpo*S(am) *rob*A1 *cre*C510 *hsd*R514 *end*A *rec*A1 *uidA(ΔMlu I)::pir-116*	[2]
HBL1	PIR1 carrying pSTV28 and pEVS104; Cm^R, Km^R	[3]
Plasmids		
pSTV28	p15Aori; Cm^R	[4]
pEVS104	oriR6K helper plasmid containing conjugal *tra* and *trb*; Km^R	[5]
pK18mobsacB	pMB1ori allelic exchange vector containing oriT; Km^R	[6]
pBBR122	Broad host range vector: Cm^R, Km^R	[7]

[a] [1] Kikuchi et al. (2011) ISME J 5, 446-460; [2] Invitrogen; [3] Kim et al. (2013) PNAS; [4] Takara; [5] Stabb and Ruby (2002) Methods Enzymol 358, 413-426; [6] Schäfer et al. (1994) J Bacteriol 176, 7309-7319; [7] Szpirer et al. (2001) J Bacteriol 183, 2101-2110.

Table S2. PCR primers used in this study.

PCR target region or purpose	Primer name	Sequence (5’-3’)
treY qPCR	treY-qPCR-P1	GTTTCCGGTGTATCGCATCT
	treY-qPCR-P2	CTGATCGAGCACGACATGAT
treZ qPCR	treZ-qPCR-P1	CGAAGGCTTTGCGTATCAG
	treZ-qPCR-P2	TCTGCAGGAACATGACGAAG
otsA qPCR	otsA-qPCR-P1	AATCTGGTGGCGAAGGAATA
	otsA-qPCR-P2	AGATCGAACGGATTGACGAC
otsB qPCR	otsB-qPCR-P1	CTACGAGATCAAGCCGAAGG
	otsB-qPCR-P2	AGATCGTCGCCGATAAACAC
treS qPCR	treS-pPCR-P1	GGCTCAACGAGCTCAACTTC
	treS-pPCR-P2	GCTTCAGCACGATCTTGTCC
recA qPCR	recA-qPCR-P1	GTCGAAGACATCCAGGTGGT
	recA-qPCR-P2	ACTCCGGACCGTAGATTTCC
5’ region of *otsA*	otsA-L-P1	cccGGATCCAGGGATTACAACGGATGACG
	otsA -L-P2	cccTCTAGACATGTAGCCCGCGTATTCTT
3’ region of *otsA*	otsA -R-P1	cccTCTAGA TTCGATCTGTCGCAGATGTC
	otsA -R-P2	cccAAGCTT GGAGAACATCAGGCAATGGT
5’ region of *treS*	treS-L-P1	cccGGATCCTCCCAACAGCGAGGAATATC
	treS-L-P2	cccTCTAGAATAGAACGGCAAGAGCCAGA
3’ region of *treS*	treS-R-P1	cccTCTAGAGTCATCCTGCAGGGCTACAT
	treS-R-P2	cccAAGCTTCGCTACGAGTGTTCATGGTG
otsA deletion check	otsA -up	CGTTAGACGATGCCCAGAAT
	otsA -down	CTGATTTCGTCGAAGAGACG
treS deletion check	treS-up	ATATCAACCCGCGTTTCTTG
	treS-down	GATGTCGAGCGGATTGAGTC
otsA complemented strain	otsA-com-P1	GGCACAGCTTTTGCATACCC
	otsA-com-P2	GGTTCGTGTCCATGACGAGT
treS complemented strain	treS-com-P1	GCACACCAATAACAAGGGCG
	treS-com-P2	GTCGATGTCGAGCGGATTGA

Figure S1. Surfactant (SDS) (**A**) and oxidative stress (H_2O_2) (**B**) assays of wild-type, Δ*otsA*, and Δ*treS* strains of *B. insecticola*. Bacterial growth inhibition by surfactant and oxidative stress is indicated by clear halos. The similar diameters of clear halos among strains indicate no difference in susceptibility to SDS or H_2O_2.

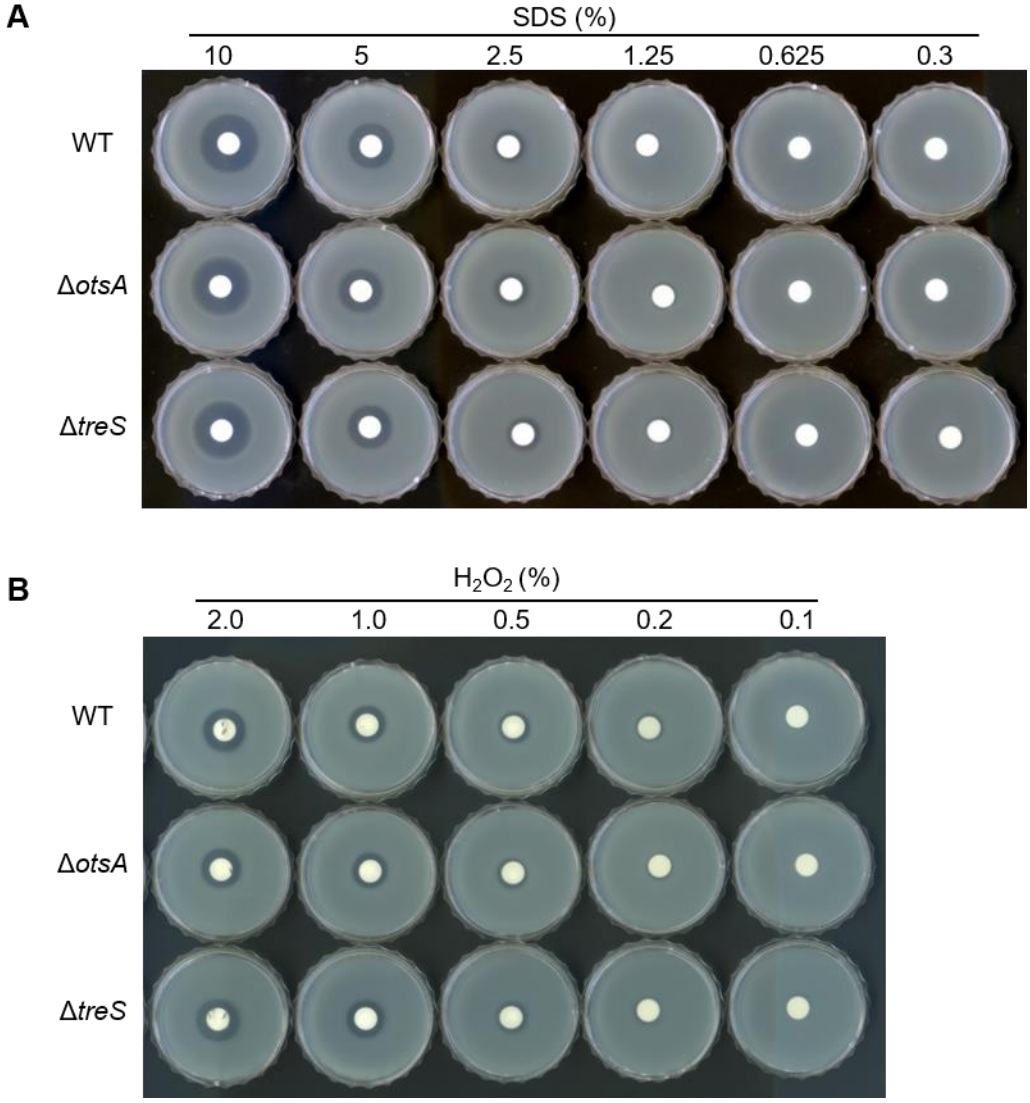

Figure S2. Motility and biofilm assays of wild-type, Δ*otsA*, and Δ*treS* strains of *B. insecticola*. (**A**) Similar bacterial motilities among strains are indicated by the opaque halos with similar diameters. (**B**) Biofilm formation was quantified by measuring the OD_{540} of dissolved crystal violet divided by the bacterial OD_{600}. Means and SDs (n = 10) are shown as columns and error bars, respectively. The same letter (a) on the top of columns indicates statistically insignificant difference (one-way ANOVA with Tukey's multiple-comparison test).

A

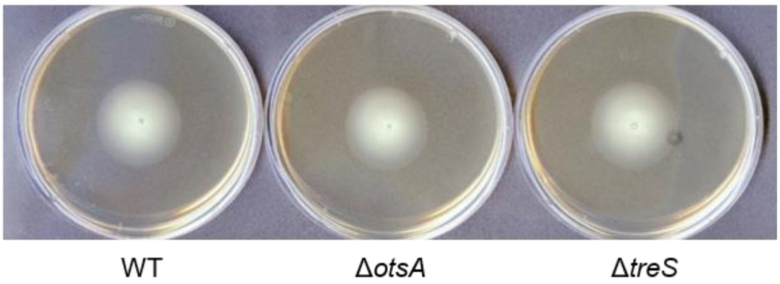

B

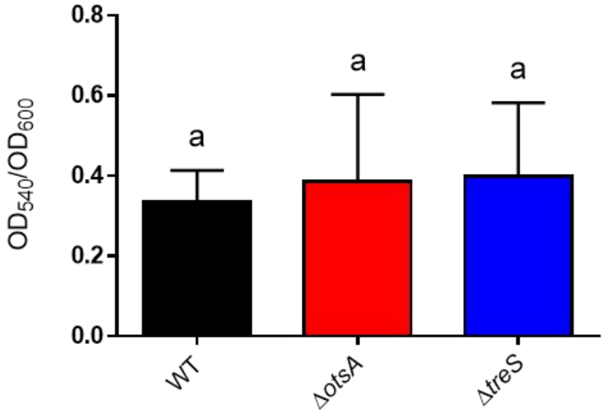

Figure S3. Trehalose biosynthetic gene expression in cultured *Burkholderia* cells under stress conditions. Means and standard deviations (SDs) (n = 3) are shown as columns and error bars, respectively. Asterisks indicate statistically significant differences (multiple t tests with Holm-Sidak methods; * $P < 0.5$, ** $P < 0.05$, *** $P < 0.005$, **** $P < 0.0005$).

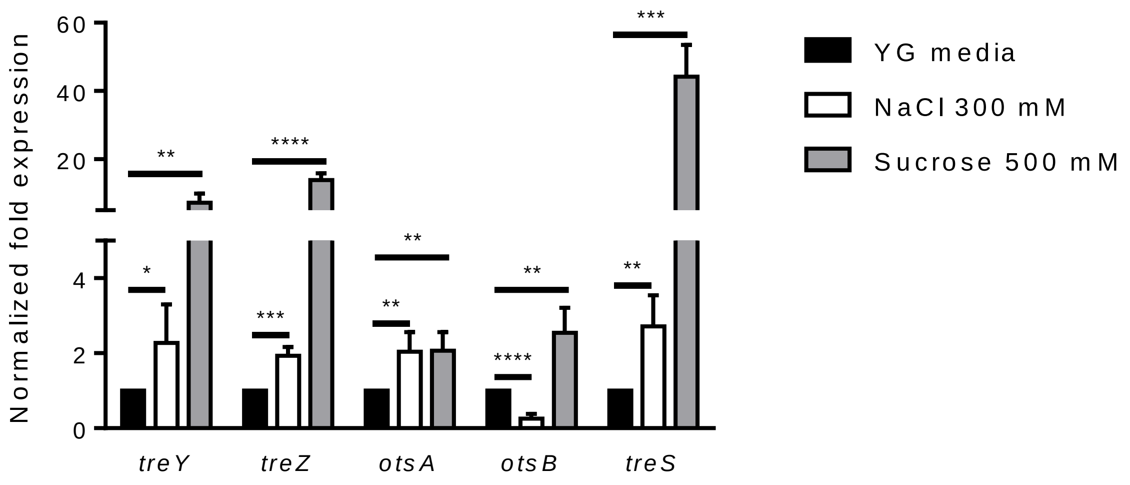

Figure S4. Effects of wild-type, Δ*otsA*, and Δ*treS* strains of *B. insecticola* on the host. (**A**) Effect of *B. insecticola* strains to host growth was measured by adult emergence rates of symbiotic insect with wild-type strain (Sym-WT), symbiotic insect with Δ*otsA* strain (Sym- Δ*otsA*), symbiotic insect with Δ*treS* strain (Sym- Δ*treS*), and aposymbiotic insect without *Burkholderia* symbiont (Apo). (**B**) Dry weight of early adult insects were measured as a representative fitness parameter of host bean bugs. Means and SDs are shown. Different letters (a, b, c) above bars indicate statistically significant differences among experimental groups (one-way ANOVA with Tukey's multiple-comparison test; $P < 0.05$).

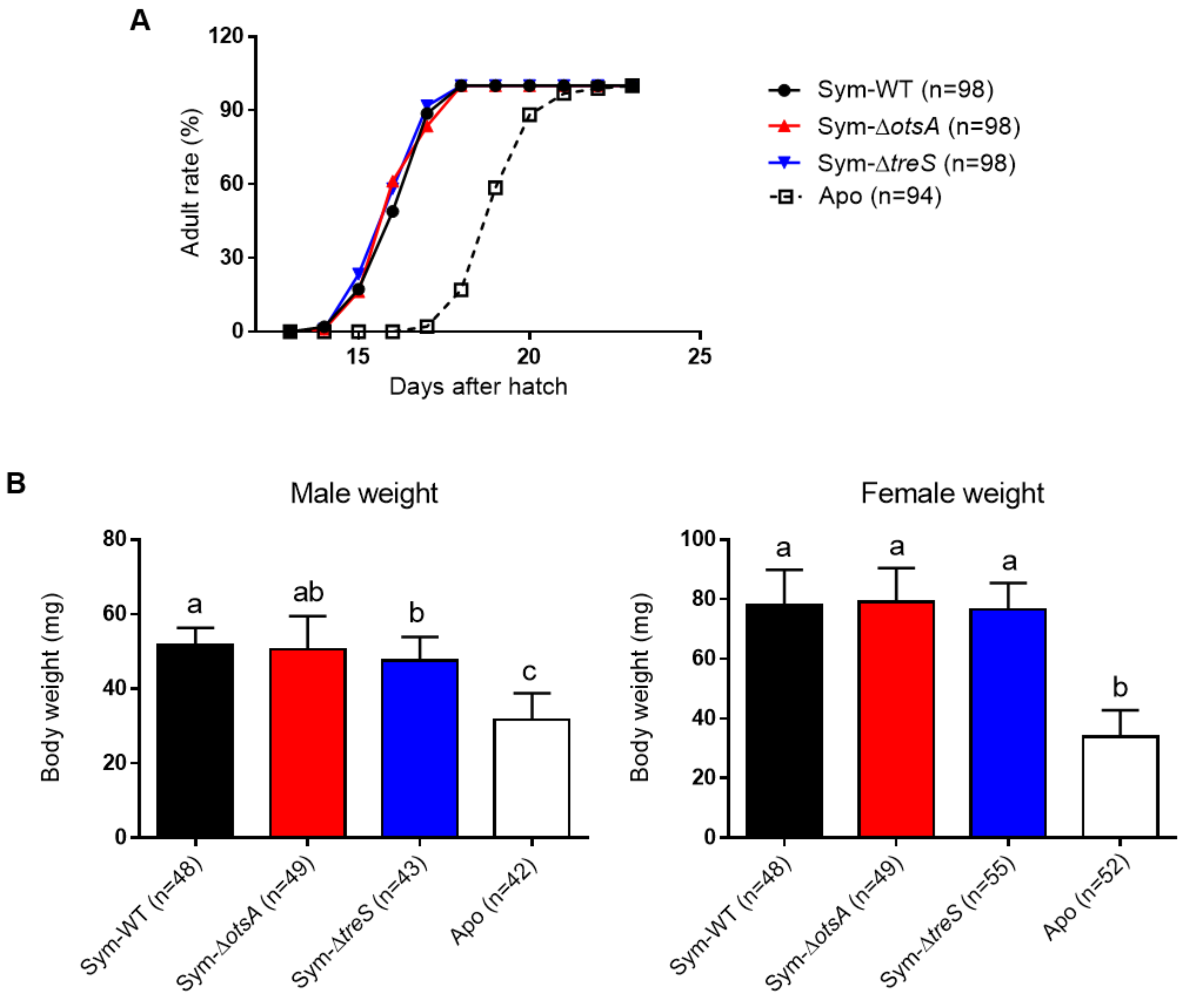

Supplement: Supplemental file 1 — Supplemental material. Download spectrum.03510-22-s0001.pdf, PDF file, 0.5 MB [file spectrum.03510-22-s0001.pdf]
